# Articular cartilage and meniscus reveal higher friction in swing phase than in stance phase under dynamic gait conditions

**DOI:** 10.1038/s41598-019-42254-2

**Published:** 2019-04-08

**Authors:** Daniela Warnecke, Maxi Meßemer, Luisa de Roy, Svenja Stein, Cristina Gentilini, Robert Walker, Nick Skaer, Anita Ignatius, Lutz Dürselen

**Affiliations:** 1 Institute of Orthopaedic Research and Biomechanics, Centre for Trauma Research Ulm, Ulm University Medical Centre, Ulm, Germany; 2 Orthox Ltd., Abingdon, UK

## Abstract

Most previous studies investigated the remarkably low and complex friction properties of meniscus and cartilage under constant loading and motion conditions. However, both load and relative velocity within the knee joint vary considerably during physiological activities. Hence, the question arises how friction of both tissues is affected by physiological testing conditions occurring during gait. As friction properties are of major importance for meniscal replacement devices, the influence of these simulated physiological testing conditions was additionally tested for a potential meniscal implant biomaterial. Using a dynamic friction testing device, three different friction tests were conducted to investigate the influence of either just varying the motion conditions or the normal load and also to replicate the physiological gait conditions. It could be shown for the first time that the friction coefficient during swing phase was statistically higher than during stance phase when varying both loading and motion conditions according to the physiological gait pattern. Further, the friction properties of the exemplary biomaterial were also higher, when tested under dynamic gait parameters compared to static conditions, which may suggest that static conditions can underestimate the friction coefficient rather than reflecting the *in vivo* performance.

## Introduction

The fibro-cartilaginous menisci play a decisive role within the knee joint. Due to its semi-lunar shape and wedge-shape cross section, it increases the contact area between the incongruent articulating surfaces of femur and tibia, thereby homogenising the load distribution within the joint^1–4^. Additionally, it is involved in joint stabilisation, nutrient distribution and lubrication^1–3,5,6^. Due to the high loads up to 3 times bodyweight (BW)^7,8^, which are transmitted through the menisci, it is prone to injuries. Here, the gold standard therapy is still a (partial) meniscectomy, although it has been shown that this can lead to cartilage degeneration in the long-term due to both an increase in contact pressure and a greater friction^9–13^. Consequently, there is an increased need for treatment strategies to restore and/or replace the meniscus. Among different research approaches, it is not yet possible to replace meniscal tissue by a material that exhibits both satisfying mechanical and tribological performance^14–16^. Here, it is stated in the literature that the tribological properties should mimic that of the native tissue as close as possible, thereby friction coefficients less than 0.05 are desirable for a well-functioning replacement material^17^. We recently reported friction coefficients of around 0.056 of a silk fibroin scaffold for partial meniscal replacement, which is in the range of the requirements for meniscal replacements postulated by Rongen *et al*.^17,18^.

The knee as a synovial/diarthrodial joint is a complex biological and mechanical system, which allows articulation and movement over millions of load cycles during a lifespan of more than 80 years^19^. This is granted by unique lubrication mechanisms provided by articular cartilage, menisci and synovial fluid and their special biphasic ultrastructure^1,3,4,20–23^. In general, meniscus and cartilage consist of a fluid (water; 70–85%) and a solid phase, which is composed of a highly specialized extracellular matrix in each of these tissues^1^. Both native forms of the tissues exhibit remarkably low friction coefficients of partly less than 0.01^12,18,23,24^, which are, however, complex as they depend on a variety of parameters, like a variation over time, lubricant, sliding velocity, applied normal load and opposing surface^19,25–27^. Nevertheless, most previous studies investigated cartilage and meniscus friction under constant normal loading conditions and sliding velocities ranging from 0.02–4 MPa and 0.1–50 mm/s, respectively^18,23,25,26,28,29^. Based on these static testing conditions and on the three lubrication modes (boundary-, mixed- and fluid lubrication), tribological theories were postulated to describe the low friction properties^19,28–33^. But taking into account that during gait the tibiofemoral contact loads acting parallel to the tibial axis (axial load)^34^ as well as the velocity of femoral and tibial surfaces relative to each other vary considerably^35–37^, it is obvious that the testing conditions used so far do not reflect the conditions typically occurring *in vivo*^18,19,28–33^. In general, a gait cycle of one leg can be divided into a stance phase (60%) initiated by heel strike and terminated by toe-off and a swing phase (40%), respectively. The tibiofemoral contact forces differ considerably between both phases. While a double-peak loading characteristic of 2–3 times BW occur within stance phase, the loads during swing phase are much lower^34,38,39^. Simultaneously, the surfaces of femur, meniscus and tibia move relative to each other. During stance phase the knee flexion angle increases from 0° at heel strike to a maximum of 15°, while during swing phase the flexion angle rises to approximately 60°^34–38^. This results in relative velocities between the articulating surfaces of 150 mm/s in average during stance- and up to 300 mm/s during swing phase^19,35^, which is far beyond the velocities that were typically used in previous friction studies. To the best of our knowledge, there are only two studies assessing the friction of the physiologically articulating surfaces and a meniscal replacement material under sinusoidal^27^ or simulated physiological loading conditions of the knee joint, respectively^16^. However, both used constant sliding velocities of 1 mm/s^27^ and 4 mm/s^16^, which were not in the range of physiological velocities in the knee joint^35,36^. Consequently, there is a lack of information in literature regarding the influence of both continuously varying the sliding velocity and simultaneously varying loading and motion conditions according to a gait cycle on friction coefficient of articular surfaces. Thus, the aim of this study was first to investigate the friction properties of the articulating surfaces within the knee joint – meniscus and articular cartilage - under testing conditions characteristically occurring within the joint during walking and second, to examine the influence of these simulated physiological testing conditions also on a potential biomaterial for meniscal replacement and therefore making possible predictions regarding its chondroprotective effect *in vivo*. Therefore, a dynamic friction testing device was developed in a *pin-on-plate* testing configuration applying normal, gait-related loading and motion conditions derived from stance- and swing phase to material pairings of articular cartilage, meniscus and a silk fibroin based hydrogel scaffold. To quantify the friction properties, the friction coefficient *µ* was identified throughout the tests.

## Material and Methods

### Sample preparation

Ten fresh bovine knee joints were ordered from a local butcher and frozen at −20 °C until the day before testing. After thawing for 1 day at 4 °C, the knees joints were examined in terms of integrity and dissected according to our standard protocol. Cylindrical meniscus and cartilage as well as the flat cartilage samples were harvested out of each knee joint as previously described within the static friction study of the silk fibroin scaffold using a trephine drill or a biopsy punch (Ø = 6 mm) and a peeler, respectively^18^. As an additional testing material, ten cylindrical samples were punched out of flat sheets (initial height: 4.9 ± 0.2 mm) of material for meniscal replacement (FibroFix Meniscus, Orthox Ltd.) using a 6 mm biopsy punch, as well.

### Dynamic friction testing device

**Figure 1:**
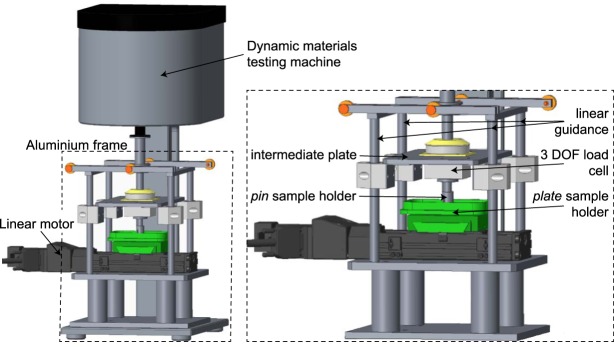
Dynamic friction testing device consisting of a dynamic materials testing machine (ElectroForce 5500, including a 1 DOF load cell, 200 N, accuracy class ≤ 1%, WMC-50-456, both BOSE/TA Instruments, New Castle, USA) equipped with a linear motor (linear stage VT-75, PI miCos GmbH, Eschbach, Germany), which was mounted on an additional aluminium frame (left). This frame was designed out of four linear guidance, an intermediate *plate*, a ball cushion (not shown in detail), the* pin* sample holder, a second load cell for measuring the resultant friction force *F*_*F*_ (3 DOF, maximum *F*_*x,y*_ = 20 N, maximum *F*_*z*_ = 50 N; accuracy class: 0.5%; ME Meßsysteme GmbH, Henningsdorf, Germany) (right) and additional counter weights (not shown).

To investigate the frictional behaviour of the different material pairings under physiological testing conditions, a dynamic materials testing machine (ElectroForce 5500, including a 1 DOF load cell, 200 N, accuracy class ≤ 1%, WMC-50-456, both BOSE/TA Instruments, New Castle, USA) was equipped with a linear motor (linear stage VT-75, PI miCos GmbH, Eschbach, Germany) mounted on a customized aluminium frame (Fig. 1). The aluminium frame comprised four linear guidances, an intermediate plate, a ball cushion, a *pin* sample holder and a second load cell for measuring the resultant friction force *F*_*F*_ (3 DOF, maximum *F*_*x,y*_ = 20 N, maximum *F*_*z*_ = 50 N; accuracy class: 0.5%; ME-Meßsysteme GmbH, Henningsdorf, Germany). Additional counter weights were installed to prevent any load application to the *pin* due to the tare weight of the frame. The linear motor, carrying the flat cartilage sample within a sample well, moved the *plate* sample holder in reciprocating manner.

Next to quasi-static testing conditions, the dynamic materials testing machine provided dynamic, freely configurable load application profiles to the *pin*. Using this feature, it was possible to generate loading conditions acting in the knee joint during normal level walking at a physiological walking speed of 5 km/h. Hence, a double-peak loading regime was applied representing the stance phase (*p*_*max,1*_ ≅ 0.9 MPa, *p*_*max,2*_ ≅ 0.8 MPa) followed by a low load plateau (*p* ≅ 0.2 MPa) simulating the swing phase in the knee joint^34^. Simultaneously, the stage motor, driven in a position controlled mode, followed up the distances that were ran over during both phases of a gait cycle in a defined period of time of 1.1 s. The input data were defined by assuming a constant radius of the femoral condyles of *r* = 25 mm as well as 15° and 60° as the maximum flexion angles during stance and swing phase, respectively. The resultant stroke lengths of 6 mm for stance- and 25 mm for swing phase were calculated using the radian measure (1).

1 $$b=\pi r\frac{\alpha }{180}$$

To ensure that both actuators, the linear motor and dynamic materials testing machine, were moving synchronously, every simulated gait cycle a trigger signal was send by the dynamic materials testing machine to a custom-made LabVIEW program (LabVIEW, National Instruments, Austin, USA). This software was developed to control the linear motor and processes these signals for data acquisition, whereby the applied normal force *F*_*N*_ and the resultant friction force *F*_*F*_ were continuously recorded (sample rate: 100 Hz) to determine the friction coefficient *µ* (2).

2 $$\mu =\frac{{F}_{F}}{{F}_{N}}$$

### Testing protocol

**Figure 2:**
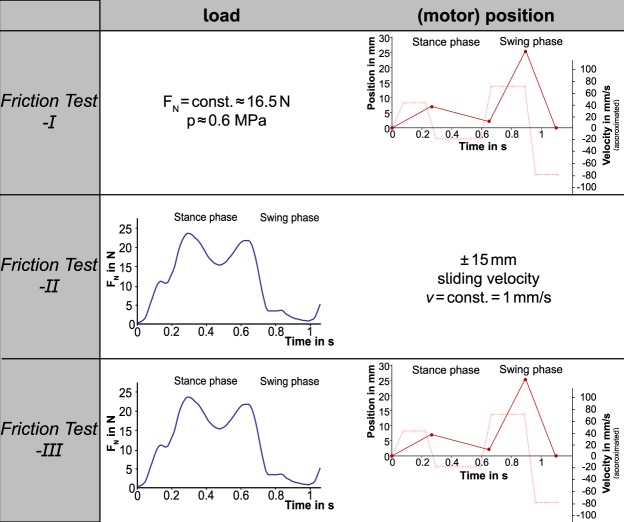
Overview of the three friction test scenarios and the resultant applications of load (*F*_*N*_) and motion: (motor) position and the approximated velocity in mm/s.

As the current study is the first synchronously applying loading and motion conditions typically occurring in the knee joint during stance- and swing phase to the mentioned friction pairings, the influence of just varying normal load *F*_*N*_ or velocity *v* on their friction properties should be additionally addressed. Therefore, the testing protocol was divided into three test scenarios (FT-I, -II, -III) conducted on three consecutive days (Fig. 2).

Based on the literature showing a decrease in the friction coefficient of articular cartilage when the testing velocity exceeds 50 mm/s^14,26^, on the first experimental day, each cylindrical sample (meniscus M, tibial cartilage TC and FibroFix Meniscus scaffold S) was tested against the corresponding flat femoral cartilage sample (FC) under constant axial loading conditions (*p* = 0.5–0.6 MPa, acc. to^18^) and varying velocities according to stance- and swing phase of a human gait cycle with a physiological walking speed of 5 km/h (Fig. 2: FT-I).

To validate the dynamic friction testing device with the literature especially with the two studies investigating cartilage and/or meniscus friction as well as the friction properties of a potential material for meniscal replacement^16,27^, a second friction test (FT-II) was added to the testing protocol. Here, the sliding velocity of the *plate* was kept constant (1 mm/s) as previously done^18^ and the load application to the *pin* (*F*_*N*_) varied cyclically according to the double-peak loading regime acting during stance phase followed by a low plateau simulating the swing phase of a gait cycle (Fig. 2: FT-II).

The third friction test (FT-III) combined both dynamic load and motion application to test the material pairings under conditions best resembling normal gait (Fig. 2: FT-III).

This resulted in a total of three tests per friction pairing (e.g. TC1/M1/S1 vs. FC1) each with a testing duration set to 20 minutes. Throughout the whole testing period, special attention was paid that all samples had the same recovery time without any load application once between each test within a test scenario (FT-I, -II, -III) but also between the test scenarios themselves (>12 h in PBS at 4 °C). The tests were performed at room temperature of approximately 24 °C and a humidity of approximately 21%. Ovine synovial fluid aspired from skeletally healthy knee joints directly after slaughtering, served as a lubricant. Throughout the testing period, care was taken that the samples were fully covered with lubricant.

### Statistics

The friction coefficient *µ* (*µ* = *F*_*F*_*/F*_*N*_) was determined at the onset (*µ*_0_) and at the end of the testing duration of 20 minutes (*µ*_*end*_) using a customized MATLAB script. (MATLAB R2013b, The MathWorks Inc., Natick, USA,). Therefore, µ of the first and last three simulated gait cycles were averaged for *µ*_0_ and *µ*_*end*_, respectively, each additionally separated for stance- and swing phase.

Based on the previous static friction study^18^, a power analysis was performed to detect differences in the friction coefficient between the friction pairings (M, TC, S vs. FC) using G*Power^40^. A total sample size of 5 was calculated to get an actual power of 0.99. Due to the complexity of the defined testing protocol, the maximum calculated samples size was doubled leading to a final total samples size of n = 10.

All further statistical analyses were performed using GraphPad Prism® software (GraphPad Software Inc., La Jolla, USA).

1. The effect of the testing duration on the friction coefficient (here the comparison of *µ*_0_ and *µ*_*end*_) of the different friction pairings (M, TC, S vs. FC) within each specific test scenario (FT-I, -II, -III), were evaluated using repeated measures one-way Analyses of Variances (ANOVA) with Sidak’s post hoc test for multiple comparison, if the data were normally distributed. Otherwise, the nonparametric Friedman test with Dunn’s post hoc test for multiple comparison were conducted.
2. To determine differences in the friction coefficient of stance- and swing phase due to the different load patterns in the test scenarios (FT-I vs. FT-II vs. FT-III) for each friction pairing (M, TC, S vs. FC), one-way ANOVAs with Sidak’s post hoc test for multiple comparison were performed, if the data were normally distributed. Otherwise, the nonparametric Friedman test with Dunn’s post hoc test for multiple comparison were conducted.
3. To compare the friction coefficients of stance- and swing phase between the friction pairings (M, TC, S vs. FC) for each test scenario (FT-I, -II, -III), mixed-effects analysis (REML) with Tukey’s post hoc test for multiple comparison were accomplished.

The statistical significance level was set to p < 0.05.

## Results

A summary of all friction coefficients (*µ*_0_ and *µ*_*end*_) obtained during the three test scenarios (FT-I, -II, -III) separated for both phases of a gait cycle, stance- and swing phase as well as for the friction pairings: tibial cartilage (TC), meniscus (M) and the silk fibroin scaffold (S) each against a flat, femoral cartilage sample (FC) are given in Table 1 as mean ± standard deviation (SD). Here, the three different test scenarios were established to determine the influence of only varying the sliding velocity (FT-I) or normal force *F*_*N*_ (FT-II) according to the motion and loading conditions during gait, and finally the combination of both as the most physiological friction test (FT-III).

**Table 1:** Summary of all friction coefficients (mean ± standard deviation) obtained during the three different test scenarios: FT-I (*F*_*N*_ = const., *v* acc. to gait cycle), -II (*F*_*N*_ acc. to gait cycle, *v* = const.) and –III (*F*_*N*_ and *v* acc. to gait cycle) for the friction pairings: tibial cartilage (TC), meniscus (M) and the silk fibroin scaffold (S) each against a flat, femoral cartilage sample (FC).

FT-I	TC vs. FC		M vs. FC		S vs. FC	
	Stance- &	swing phase	Stance- &	swing phase	Stance- &	swing phase
*µ*_0_	0.022 ± 0.012	0.025 ± 0.010	0.019 ± 0.008	0.017 ± 0.005	0.034 ± 0.013	0.036 ± 0.014
*µ*_end_	0.018 ± 0.005	0.024 ± 0.009	0.020 ± 0.006	0.017 ± 0.006	0.036 ± 0.011	0.038 ± 0.009
**FT-II**						
*µ*_0_	0.021 ± 0.013	0.027 ± 0.018	0.026 ± 0.024	0.030 ± 0.028	0.077 ± 0.041	0.122 ± 0.058
*µ*_end_	0.013 ± 0.010	0.019 ± 0.021	0.015 ± 0.010	0.017 ± 0.012	0.061 ± 0.034	0.092 ± 0.046
**FT-III**						
*µ*_0_	0.018 ± 0.005	0.032 ± 0.013	0.015 ± 0.009	0.033 ± 0.007	0.042 ± 0.017	0.043 ± 0.021
*µ*_end_	0.019 ± 0.005	0.029 ± 0.009	0.016 ± 0.007	0.030 ± 0.008	0.057 ± 0.019	0.047 ± 0.020

**Figure 3:**
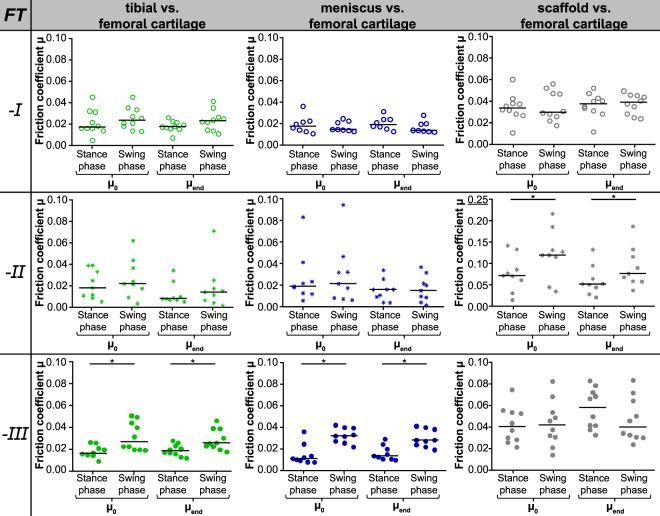
Comparison of the friction coefficients (median with raw data) for each material pairing (M, TC, S vs. FC, divided by column) obtained in the three different friction test scenarios (FT-I: *F*_*N*_ = const., *v* acc. to gait cycle, FT-II: *F*_*N*_ acc. to gait cycle, *v* = const., FT-III: *F*_*N*_ and *v* acc. to gait cycle, divided by rows). *p ≤ 0.05 with a minimum actual power of 70.1% (FT-II scaffold vs. femoral cartilage).

The evaluation of the fiction coefficient revealed no time-depended differences (*µ*_0_ vs. *µ*_*end*_) for each material pairing (M, TC or S vs. FC). This was also true for each of the three test scenarios (FT-I, -II, -III; Fig. 3). Consequently, all other analyses and comparisons were performed using the friction coefficient determined after 20 minutes testing (*µ*_*end*_).

**Figure 4:**
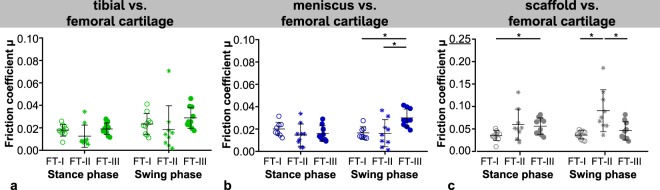
Comparison of the friction coefficients (*µ*_*end*_) obtained for each material pairing: tibial cartilage (**a**), meniscus (**b**) and scaffold (**c**) each against femoral cartilage within the three different friction test scenarios (n = 8–10, mean ± standard deviation and raw data; ○ FT-I, *FT-II, • FT-III), *p ≤ 0.05 with a minimum actual power of 96.1% and 73.1% for the comparisons the meniscus and scaffold friction coefficient, respectively.

No differences between the friction coefficients obtained during simulated stance- and swing phase could be found for both cartilaginous tissues, meniscus and tibial cartilage, each tested against flat cartilage samples when varying only the velocity (FT-I) or normal load (FT-II). Interestingly, this changed as soon as both testing parameters synchronously varied as it occurs during a physiological gait cycle (FT-III). Here, the simulated low-loaded swing phase revealed significantly higher friction coefficients than the stance phase (Fig. 3, left and central column). Additionally, the friction coefficient of meniscus against cartilage (M vs. FC) was highest for FT-III (0.030 ± 0.008) during swing phase in comparison to the other two load scenarios (FT-I and –II, 0.017 ± 0.006 and 0.017 ± 0.012, respectively), while during stance phase no differences in friction could be found for each of the three different test scenarios (p ≤ 0.05; Fig. 4b). However, the cartilage against cartilage pairing remained in general uninfluenced by the different load scenarios for both, stance- and swing phase (Fig. 4a). The silk fibroin scaffold tested against cartilage showed in general higher friction coefficients under FT-II conditions (averaged stance phase: *µ* = 0.069 ± 0.011 and swing phase: *µ* = 0.107 ± 0.021; Fig. 3, right column), which was additionally statistically significant in comparison to FT-I and FT-III (0.038 ± 0.009 and 0.047 ± 0.020, respectively) during swing phase (Fig. 4c).

**Figure 5:**
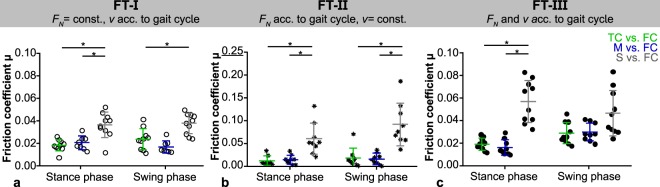
Comparison of the friction coefficients (*µ*_*end*_) obtained within each friction test scenarios (○ FT-I: A, *FT-II: B, • FT-III: C) for the different material pairings (TC, M, S vs. FC; n = 8–10, mean ± standard deviation and raw data) *p ≤ 0.05 with an actual power of approximately 99%.

Testing the material pairings either under constant loads but varying velocities (FT-I) or inversely varying the normal forces *F*_*N*_ according to the loading conditions during normal walking at 5 km/h but maintaining a constant velocity (1 mm/s, FT-II), the silk fibroin scaffold revealed the highest friction coefficients in comparison to tibial cartilage- and meniscus samples, for both, stance- and swing phase, respectively (Fig. 5a,b). This was also true during stance phase when testing under simulated physiological loading and motion conditions (FT-III, Fig. 5c). Even though, the scaffold showed a higher friction coefficient by tendency also during swing phase, no statistical differences were detected. The friction coefficient of meniscus and tibial cartilage each against femoral cartilage did not differ statistically during all three test scenarios.

## Discussion

For the first time we were able to assess friction coefficients of the articulating surfaces of the knee joint, meniscus and articular cartilage, under simulated physiological loading and motion conditions occurring during normal walking. Additionally, a silk fibroin scaffold was used for testing to investigate the influence of these new testing conditions on a potential material for meniscal replacement.

When tested under physiological testing conditions (FT-III), the friction coefficients for both cartilaginous tissues, tibial cartilage and meniscus, each tested against cartilage (TC/M vs. FC) were higher during the low-loaded swing phase (TC vs. FC: 0.029 ± 0.009, M vs. FC: 0.030 ± 0.008) than during the high-loaded stance phase (TC vs. FC: 0.019 ± 0.005, M vs. FC: 0.016 ± 0.007). Although this phenomenon appears contradictory, Majd *et al*. and Krishnan *et al*. showed an increase in friction within low-loaded phases, as well^16,27^. Krishnan *et al*. simultaneously detected negative values of the fluid load support *W*^*P*^*/W* of less than −1.75^27^ and consequently made the assumption that suction might occur between the cartilage and the counter glass platens^16,27^. This additionally led to an increased solid-to-solid contact force, resulting in higher friction coefficient, although the applied normal force is smallest^27^. Thus, once the load is rapidly decreased, the contact between the loaded cartilage-*pin* and glass after a long load application might lead to a sticking of the cartilage to the glass *plate*. Even if in both studies (inter alia) an impermeable opposing surface (e.g. glass) was used^16,27^, this phenomenon could also be observed with flat cartilage samples as counterpart during the current study especially in FT-III (and FT-II for S vs. FC), when dynamically varying the axial load. Despite the fact that Krishnan *et al*. and Majd *et al*. applied a constant velocity of 1 mm/s and 4 mm/s^16,27^, respectively, which is far below the surface velocities in the knee joint of 50–300 mm/s.^35^, their testing conditions compared well with our second friction test (FT-II) also carried out at 1 mm/s. Here, the silk fibroin scaffold generally showed the highest friction coefficients, which were again significantly increased within the low-loaded swing phase (0.092 ± 0.046; stance phase: 0.061 ± 0.034). This is again in line with the study of Majd *et al*. evaluating the friction properties of another potential material for meniscal repair under similar conditions. These authors found a more than 15-fold higher friction coefficient of the replacement material during swing phase than during stance phase (approximately 0.7 vs 0.04), while during swing phase *µ* of the silk fibroin based hydrogel scaffold tested in the current study was only 50% higher^16^. In consideration of the different lubricants used of Majd *et al*. and the current study (solution of PBS and different lubrication molecules vs. synovial fluid, respectively), the obtained results fit nevertheless quite well to the results of the referenced study (stance phase: 0.06 vs 0.04), which showed the validity of the testing device.

Next to the soaking effect and the resultant rise in the friction coefficient when the applied force rapidly decreases at the transition of stance- and swing phase, it is also known that a quite thick fluid film of approximately 1.6 µm can be formed during swing phase^19,35^ that is much larger than the average surface roughness of articular cartilage (R_a_ = 200 nm). Transferring these to the simulated gait conditions (FT-III) of the current study, it can be concluded that together with the assumed high Hersey number (low normal load and high velocity), hydrodynamic lubrication occurs in the swing phase^19,26^. This fluid film is subsequently squeezed out due to the rapid increase in load at ‘heel strike’ with beginning of the stance phase. Since, this load application has an impact characteristic (<0.1 s), the fluid film is pressurised but can be preserved between the deformable bearing material of meniscus and/or cartilage. Taking the identified low friction coefficient in the upcoming stance phase of <0.02 into account, elasto-hydrodynamic lubrication can be assumed as this is the lubrication mode of least friction coefficient in the Stribeck curve^19^. Throughout a stance phase of low velocity-to-load ratio, the synovial fluid still separates the articulating surfaces until ‘toe off’ and the initiation of the next swing phase. The distinct lubrication mechanisms of elasto-hydrodynamic- and hydrodynamic lubrication within the simulated stance- and swing phase can consequently be an explanation for the obtained differences in the friction coefficients between these gait phases when testing under physiological loading and motion conditions (FT-III). However, tibial cartilage samples were rather uninfluenced by the three different loading scenarios. The cartilage – cartilage friction pairing indeed showed by tendency the lowest friction coefficient of approx. *µ* = 0.013 during stance- and *µ* = 0.019 during swing phase and when testing under varying loading (FT-II) conditions, while *µ* was nearly identical during FT-I and FT-III (stance phase: p = 0.6415, swing phase: p = 0.3163). This indicates that additionally varying the velocity in a physiological range affect cartilage friction. The authors speculate that a reason might be the differences in the extracellular matrix (ECM) compositions of articular cartilage and meniscal tissue. Since with progressive duration of friction testing, the interstitial fluid of the loaded cylindrical samples (*pin*) of both tissues is squeezed out, the applied load is carried by their ECM and is therefore responsible for the friction coefficient. While the ECM of articular cartilage is composed of 5–10% wet wt. of proteoglycans (PG), meniscal tissue contains only a fifth of this^1^. Additionally, their main collagen type differ, as well: articular cartilage: 10–20% wet wt. collagen type II vs. meniscal tissue: 15–25% wet wt. collagen type I, which may alter the resistance to high velocities and consequently shear forces of the tissue^1–4^.

It was already shown that the friction coefficients of meniscus and cartilage are multifactorial depending on several parameters and operating conditions^26^ rather than being just a material constant as described within Coulomb’s friction law. Consequently, the mechanisms of the mentioned lubrication modes will significantly differ depending on the testing parameters, as well^26,41^. Although, it is important to perform friction tests under clearly defined static testing and lubrication regimes^26^, one should be aware that such data do not perfectly reflect the friction coefficients occurring *in vivo*, e.g. during gait. This is supported by the literature as there is a general consent that (elasto-)hydrodynamic- but also mixed lubrication mechanisms can synergistically contribute to the remarkably low friction properties of the joint^19,42^ as the loading and motion conditions vary considerably within a normal gait cycle.

As a potential material for meniscal replacement, a silk fibroin based hydrogel scaffold was additionally tested under the three different testing conditions (FT-I, -II and –III). In a previous study, the scaffold already showed friction coefficients of 0.056, which was higher than friction of native meniscus (*µ* = 0.021) but in the range of the requirements for meniscal replacement postulated by Rongen *et al*.^17^. Within the current study the material met these requirements again also under simulated gait conditions (FT-III: 0.057 ± 0.019 and 0.047 ± 0.020 for stance and swing phase, respectively).

Since the physiological testing conditions revealed higher friction coefficients for meniscal tissue especially within the simulated swing phase of almost 0.030, this suggests that static testing methods as reported in the literature with friction coefficients of less than 0.01 can underestimate friction coefficients rather than reflecting the complex *in vivo* performance. This might especially be important for potential replacement materials and their prediction regarding their chondroprotective effect *in vivo*.

For all three tests (FT-I, -II, -III) in general, no time-depended differences in the friction coefficient (*µ*_0_ vs. *µ*_*end*_) could be observed for each material pairing (M, TC or S vs. FC) either during stance- or during swing phase. However, this was not surprising as previous studies already showed that if the moving opposing surface (*plate*) is cartilaginous, no increase in friction will develop^18,23,43^. Consequently, the interstitial fluid pressurization was maintained in all three test scenarios as well as for all material pairings. While the *pin* was loaded throughout the whole test, the moving contact area of the flat cartilage surface (*plate*) was able to recover during the time of unloading before it was loaded again. Therefore its fluid phase supported the load during the whole testing duration and thus, the friction coefficient remained at the observed low level.

### Limitations

The friction testing device developed in the current study was designed according to a *pin-on-plate* configuration. Using this test setup, it was possible to apply loads and velocities occurring in the knee joint during normal walking. However, it is a simplification of the complex joint kinematics as the combined rolling and sliding motion coexisting during flexion and extension of the knee joint is not considered. Nevertheless, using a “rolling-gliding wear simulator” it was already shown that during rolling, and rolling with slip motion, the signs of wear were least when testing different artificial material pairings^44^. Consequently, the main part of friction occurs during sliding, which was considered within the dynamic friction testing device investigated in the current study. Nevertheless, to further take the rolling and sliding within the knee joint into account during friction analysis, a pendulum friction simulator would be an alternative test setup. The advantage of this test setup is that the entire knee joint is tested and therefore considered as one biomechanical and tribological system, preserving the physiological geometries and joint kinematics^45–47^. However, this also represents a disadvantage, since no distinction can be made between friction properties of cartilage and/or meniscus.

### Conclusion

The current study presents new insights in joint friction mechanics as it showed significantly lower friction coefficients during simulated stance- than during the low-loaded swing phase. This phenomenon was observed for meniscus and articular cartilage only when testing under conditions with varying both normal load and velocity as it appears during gait. The high velocities occurring in the swing phase may cause a transition from elasto-hydrodynamic to hydrodynamic lubrication and therefore, increased friction coefficient. Consequently, due to the multifactorial characteristics of cartilage and meniscus friction, the current study emphasizes the need of adding friction tests under physiological testing conditions to the tribological characterisation of materials relevant for joints and especially for potential meniscal or cartilage replacement materials. Thereby, the tested silk fibroin based hydrogel scaffold matched the friction coefficient as demanded in the basic requirements for meniscal replacement materials.
